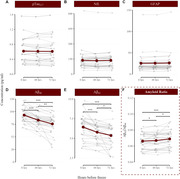# Effect of Refrigeration on Alzheimer’s Disease Biomarker Measurements in Plasma

**DOI:** 10.1002/alz.086669

**Published:** 2025-01-09

**Authors:** Ramiro Eduardo Rea Reyes, Rachael E Wilson, Elysse Keske, Martie Marshall, Cindy Jensen, Monica VandenLangenberg, Kate Lange, Beckie Jeffers, Aaron Fredricks, Victoria J. Williams, Ozioma C Okonkwo, Barbara B. Bendlin, Corinne D. Engelman, Carey E. Gleason, Kirk J. Hogan, Nathaniel A. Chin, Cynthia M. Carlsson, Erin M. Jonaitis, Sanjay Asthana, Sterling C. Johnson, Henrik Zetterberg

**Affiliations:** ^1^ University of Wisconsin‐Madison, Madison, WI USA; ^2^ University of Wisconsin, Madison, WI USA; ^3^ Alzheimer’s Disease Research Center, University of Wisconsin School of Medicine and Public Health, Madison, WI USA; ^4^ School of Medicine and Public Health, University of Wisconsin‐Madison, Madison, WI USA; ^5^ University College London, London UK; ^6^ University of Gothenburg, Gothenburg Sweden; ^7^ Hong Kong Center for Neurodegenerative Diseases, Hong Kong China

## Abstract

**Background:**

Mobile phlebotomy is an attractive option for the study of Alzheimer’s disease (AD) in rural and underserved populations, but logistics of collecting, and shipping these samples outside clinical settings can introduce additional sources of variability. Knowledge is limited on the impact of field collection on blood‐based Alzheimer’s disease biomarkers, especially protein stability when dry ice is not available during shipping. Our study looks into the effects of holding plasma samples under refrigeration prior to freezing on AD biomarker concentrations compared to samples that were frozen within one hour of collection as is standard practice.

**Method:**

We used plasma samples from 30 participants (58‐93 yo, mean age = 72.23, SD = 7.31; 18 females, 12 males) from the Wisconsin Registry for Alzheimer’s Prevention and the Wisconsin Alzheimer’s Disease Center, with aliquots from each of them undergoing three different treatments: a reference condition frozen immediately at ‐80°C, and two treatments involving refrigeration at 2‐8°C for either 48 or 72 hours before freezing. We measured the concentration of five AD blood biomarkers (ALZpath pTau_217_, GFAP, NfL, Aβ_40_, and Aβ_42_) using the Quanterix HD‐X platform. Finally, we evaluated biomarker stability by comparing refrigeration treatments to the aliquots frozen immediately, via repeated measures ANOVAs and ICC analysis.

**Result:**

We found consistent concentrations of pTau_217_ [F(1.78, 44.49) = 0.16, p = .829; ICC(3,3) = 0.987, p < 0.001], NfL [F(1.45, 39.14) = 2.84, p = .086; ICC(3,3) = 0.992, p < .001], and GFAP [F(1.63, 43.89) = 1.66, p = .205; ICC(3,3) = 0.996, p < 0.001] for up to 72 hours of refrigeration. However, both Aβ_42_ and Aβ_40_ levels in plasma significantly declined after refrigeration [Aβ_40_: F(1.22, 33.07) = 27.38, p < 0.001; ICC(3,3) = 0.537, p < .001; Aβ_42_: F(1.16, 31.34) = 15.35, p < .001; ICC(3,3) = 0.713, p < .001].

**Conclusion:**

Our results highlight the sensitivity of plasma Ab to the storage conditions, and show pTau_217_, GFAP, NfL as candidates to study when ideal handling conditions for plasma samples are not easily accessible. Their resilience under refrigeration can provide flexibility to access more remote communities and increase representation in AD research.